# Manual support during robotic-assisted percutaneous coronary intervention

**DOI:** 10.1007/s00392-025-02596-6

**Published:** 2025-02-05

**Authors:** Benjamin Bay, Alina Goßling, Jonathan Rilinger, Constantin von zur Mühlen, Felix Hofmann, Holger Nef, Helge Möllmann, Caroline Kellner, Moritz Seiffert, Fabian J. Brunner

**Affiliations:** 1 Department of Cardiology, University Heart and Vascular Center Hamburg, University Medical Center Hamburg-Eppendorf, Martinistrasse 52, 20246 Hamburg, Germany; 2 German Center for Cardiovascular Research (DZHK), Partner Site Hamburg/Kiel/Lübeck, Hamburg, Germany; 3 Center for Population Health Innovation (POINT), University Heart and Vascular Center Hamburg, University Medical Center Hamburg-Eppendorf, Hamburg, Germany; 4 Department of Cardiology and Angiology, Faculty of Medicine, University Heart Center Freiburg - Bad Krozingen, University of Freiburg, Freiburg, Germany; 5 Department of Cardiology and Angiology, University Hospital of Giessen and Marburg, Giessen, Germany; 6 German Center for Cardiovascular Research (DZHK), Partner Site Rhine-Main, Bad Nauheim, Germany; 7 Cardiology Department, Heart Center, Segeberger Kliniken GmbH, Bad Segeberg, Germany; 8 Department of Cardiology, St. Johannes Hospital, Dortmund, Germany; 9 Department of Cardiology and Angiology, BG University Hospital Bergmannsheil, Ruhr-University Bochum, Bochum, Germany

**Keywords:** Percutaneous coronary intervention, Robotics, Manual support, Predictors

## Abstract

**Background:**

Robotic-assisted percutaneous coronary intervention (R-PCI) is an efficacious and safe treatment option for coronary artery disease. However, predictors of manual support during R-PCI are unknown, which we aimed to investigate in a multi-center study.

**Methods:**

We utilized patient-level data from R-PCIs carried out from 2020 to 2022 at four sites in Germany. Manual support was defined as the combination of partial manual assistance, where the procedure is ultimately completed using robotic techniques, and manual conversion. A two-step selection process based on akaike information criteria was used to identify the ideal multivariable model predicting manual support.

**Results:**

In 210 patients (median age 69.0 years; 25.7% female), a total of 231 coronary lesions were treated by R-PCI. Manual support was needed in 46 lesions (19.9%). Procedures requiring manual support were associated with significantly longer procedural times, greater total contrast fluid volumes, longer fluoroscopy times, and higher dose-area products. Amongst the predictors of manual support were lesions in the left anterior descending artery [OR: 1.09 (95%-CI: 0.99–1.20)], aorto-ostial lesions [OR: 1.35 (95%-CI: 1.11–1.64)], chronic total occlusions [OR: 1.78 (95%-CI: 1.38–2.31)], true bifurcations [OR: 1.37 (95%-CI: 1.17–1.59)], and severe calcification [OR: 1.13 (95%-CI: 1.00–1.27)].

**Conclusion:**

Our findings reveal that nearly one out five of patients undergoing R-PCI required manual support, which was linked to longer procedure durations. Predictors of manual support reflected characteristics of more complex coronary lesions. These results highlight the limitations of current R-PCI platforms and underscore the need for technical advancements to address different clinical scenarios.

**Graphical abstract:**

Predictors for manual support during robotic-assisted percutaneous coronary intervention. 95%-CI: 95%-confidence interval, DES: Drug eluting stent, OR: Odds ratio, LAD: Left anterior descending coronary artery, R-PCI: robotic-assisted percutaneous coronary intervention.

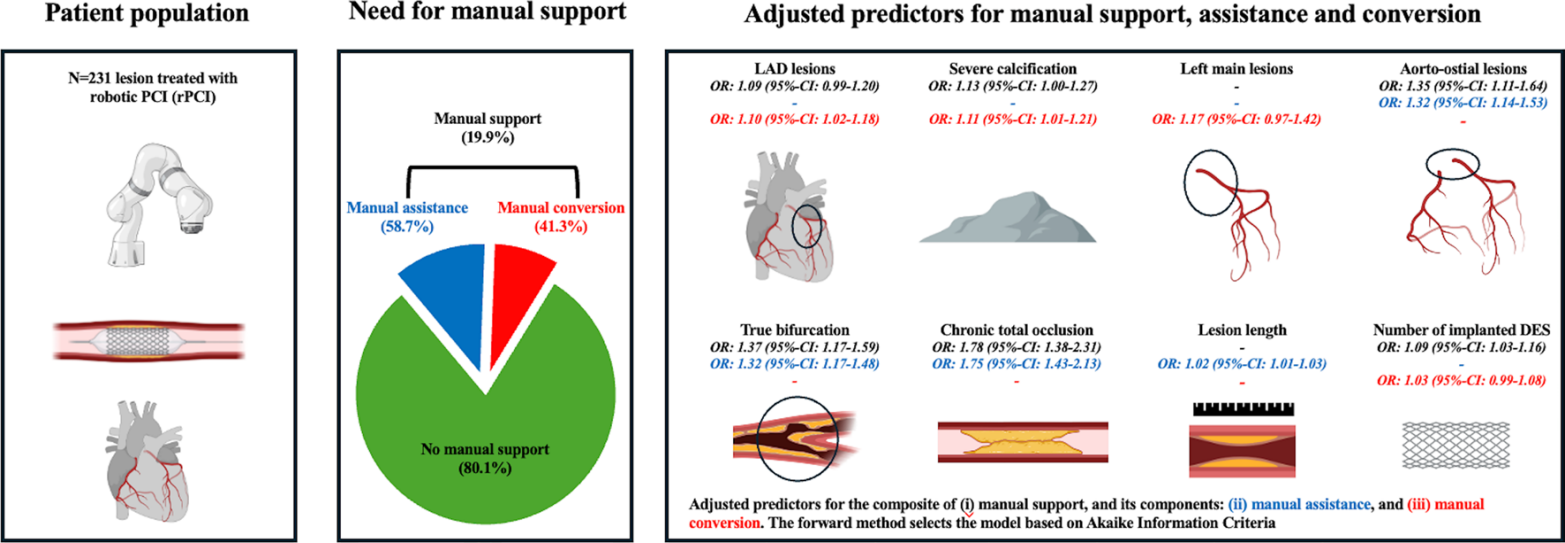

**Supplementary Information:**

The online version contains supplementary material available at 10.1007/s00392-025-02596-6.

## Introduction

Percutaneous coronary intervention (PCI) is one of the most commonly used techniques in the treatment of atherosclerotic coronary lesions and coronary artery disease (CAD) [1]. Over the last years, robotic-assisted percutaneous coronary intervention (R-PCI) has proven itself to be an efficacious and safe treatment option with similar outcomes compared to manual PCI [2–5]. Also, R-PCI has the ability to help reduce or prevent radiation exposure and musculoskeletal injuries among treating interventional cardiologists [6]. Several technological advancements have been made since the introduction of the first R-PCI platforms, allowing the treatment of more complex coronary lesions [7]. However, in certain R-PCI cases, manual support is still required to successfully complete the R-PCI procedure [8]. Potential predictors of manual support, however, have not been investigated. We, therefore, sought to identify factors affecting the need for manual support during R-PCI using the latest generation robotic PCI platform in a multi-center cohort.

## Methods

### Study cohort

For the current analysis, individual-level data from R-PCIs carried out at four interventional sites in Germany (Department of Cardiology, University Heart & Vascular Center in Hamburg-Eppendorf; Department of Cardiology and Angiology, University Heart Center Freiburg—Bad Krozingen, Faculty of Medicine, University of Freiburg; Department of Cardiology and Vascular Medicine, Medical Faculty, Justus-Liebig-University Giessen; and Department of Cardiology, St. Johannes Hospital, Dortmund) were obtained. This study was performed in accordance with the Declaration of Helsinki. All patients undergoing R-PCI from 2020 to 2022 were included in respective registries at each site. Data was routinely collected during clinical practice with no need for institutional review board nor central ethics committee approval (Dortmund) or in accordance with local ethics committee approval (Hamburg: PV4303; Freiburg: 20–1344; Giessen: 233/19).

### Robotic-assisted PCI and manual support, assistance and conversion

R-PCI was carried out using the 2nd generation CorPath GRX Vascular Robotic System (Corindus Inc., Siemens Healthineers Company, Waltham, USA). All R-PCI procedures were implemented by experienced interventional cardiologists. The treatment approach with regard to the interventional treatment of coronary lesions, peri-procedural care, and subsequent medical treatment was at the discretion of the treating interventionalist in accordance with current guidelines and institutional standards of care. Manual support was defined as the necessity of manual assistance (partial manual assistance with the procedure ultimately completed robotically) or manual conversion (bedside manipulation of the guide catheter, guidewire, or delivery system needed to complete the procedure).

### Assessment of baseline procedural and lesion characteristics

At baseline, patient characteristics including demographics, comorbidities, prior medical history and laboratory values were obtained using a questionnaire and patient charts. All analyses of the coronary angiogram including lesion classification and procedural aspects were conducted by trained interventional cardiologists. The CAD classification categorized coronary artery disease as 1-, 2-, or 3-vessel disease based on the number of major epicardial vessels with ≥ 50% diameter stenosis or a history of PCI. Lesions were classified according to the American College of Cardiology/American Heart Association (ACC/AHA) lesion morphology system in its modified version [9]. Coronary calcification was evaluated by the operator on angiography using the following classification: none–mild, moderate, or severe calcification. Angiographic success was defined as achieving residual diameter stenosis of less than 20% in the target lesion with Thrombolysis in Myocardial Infarction grade 3 flow [10]. True bifurcations were categorized based on the Bifurcation Academic Research Consortium criteria, with true bifurcations defined as lesions with ≥ 50% diameter stenosis in both the main vessel and side branch, classified as Medina 1,1,1; 1,0,1; or 0,1,1 [11].

### Statistical approach

Patients who received manual support were compared to those who underwent a complete R-PCI approach. Categorical variables are presented as absolute counts and percentages and were analyzed using Fisher's exact test. Continuous variables are expressed as either the median (with 25th and 75th percentiles) or the mean ± standard deviation (SD) and were compared using the Mann–Whitney test or *t*-test, respectively. To develop the best uni- and multivariable model predicting manual support, manual assistance and manual conversion we employed a two-step selection process based on Akaike Information Criteria. Odds ratios (OR) and their 95%-confidence intervals (95%-CI) were computed. Given the sample size, a Firth correction was added to the final multivariable regression model. Statistical significance was defined as a two-sided *p*-value of less than 0.05. All analyses were performed using R statistical software (version 4.2.1, R Foundation for Statistical Computing).

## Results

### Baseline characteristics

In 210 patients (median age 69.0 years; 25.7% female), a total of 231 coronary lesions were treated by R-PCI. Manual support was needed in 19.9% of all lesions (*n* = 46). Detailed baseline characteristics for the overall cohort, full robotic and manual support subgroup, respectively, are provided in Table 1. No differences in demographics, comorbidities, prior medial history and laboratory parameters were noted between patients with complete R-PCI and those requiring manual support. Baseline characteristics for manual support subgroups (manual assistance and manual conversion, respectively) are included in Supplementary Table S1.

**Table 1:** Baseline characteristics

	Overall (*N* = 210)	Full robotic (*N* = 167)	Manual support (*N* = 43)	*p*-value
Demographics				
Age: years	69.0 (60.2, 79.0)	69.0 (59.5, 79.0)	68.0 (61.5, 79.5)	0.76
Male sex: no. (%)	156 (74.3)	128 (76.6)	28 (65.1)	0.17
BMI: kg/m^2^	27.3 (24.5, 30.9)	27.7 (24.7, 31.2)	25.3 (23.8, 30.5)	0.062
Comorbidities and prior medical history				
Arterial hypertension: no. (%)	167 (79.5)	130 (77.8)	37 (86.0)	0.29
Diabetes mellitus: no. (%)	84 (40.0)	71 (42.5)	13 (30.2)	0.16
Current smoking: no. (%)	29 (20.3)	23 (20.7)	6 (18.8)	1.00
History of smoking: no. (%)	114 (55.1)	88 (53.7)	26 (60.5)	0.49
LVEF: %	55.0 (40.2, 60.0)	55.0 (42.5, 60.0)	50.0 (37.2, 55.0)	0.10
History of cerebrovascular disease: no. (%)	40 (19.0)	29 (17.4)	11 (25.6)	0.28
History of PAD: no. (%)	24 (11.4)	20 (12.0)	4 (9.3)	0.79
History of CABG: no. (%)	17 (8.1)	12 (7.2)	5 (11.6)	0.35
History of PCI: no. (%)	132 (62.9)	106 (63.5)	26 (60.5)	0.73
History of myocardial infarction: no. (%)	74 (35.2)	58 (34.7)	16 (37.2)	0.86
Laboratory values				
LDL-C: mg/dL	91.0 (63.2, 116.0)	93.0 (63.0, 118.0)	77.0 (67.0, 111.0)	0.35
Serum creatinine: mg/dL	1.0 (0.8, 1.3)	1.0 (0.9, 1.3)	0.9 (0.8, 1.3)	0.23
eGFR: ml/min/1.73m^2^	72.7 (53.7, 89.0)	72.0 (53.6, 89.2)	75.0 (55.9, 89.0)	0.72

Categorical variables are presented as counts with corresponding percentages, while continuous variables are summarized using the mean and standard deviation (SD) or the median along with the 25th and 75th percentiles

*BMI* body mass index, *eGFR* estimated glomerular filtration rate, *LDL-C* low-density lipoprotein cholesterol, *LVEF* left ventricular ejection fraction, *PAD* peripheral arterial disease, *PCI* percutaneous coronary intervention

### Lesion characteristics

Characteristics of all 231 coronary lesions treated by R-PCI are displayed in Table 2. In both groups a trans-radial access was used in the majority of cases (75.7% vs. 73.9% for complete R-PCI vs. manual support; *p*-value = 0.85). No between group differences were noted with regard to the extent of CAD reflected by the number of narrowed major epicardial coronary vessels. The left anterior descending artery (LAD) was intervened most frequently (35.1% in the full robotic group vs. 47.8% in the manual support group; *p*-value = 0.13) followed by the right coronary artery (33.5% vs. 26.1%, *p* = 0.38) and circumflex coronary artery (26.5% vs. 28.3%, *p* = 0.85). A higher prevalence of complex lesions such as aorto-ostial lesions (3.8% vs. 15.2%; *p*-value = 0.0091), chronic total occlusions (CTOs; 0.5% vs. 17.4%; *p*-value < 0.001) and true bifurcations (7.7% vs. 26.1%; *p*-value = 0.0012) was noted in the manual support group. From the included CTOs, eight out of nine were treated with manual support. Moreover, a higher proportion of Type C lesions as defined using the AHA/ACC criteria (41.6% vs. 78.3%; *p*-value < 0.001), longer overall lesion length (15.0 vs. 23.7 mm; *p*-value = 0.0067) and a higher proportion of severely calcified lesions (15.1% vs. 34.8%; *p*-value = 0.0054) was recorded in those patients requiring manual support. Lesion characteristics stratified according to manual assistance and manual conversion, respectively, are included in Supplementary Table S2.

**Table 2:** Lesion characteristics

	Overall (*N* = 231)	Full robotic (*N* = 185)	Manual support (*N* = 46)	*p*-value
Radial access site: no. (%)	174 (75.3)	140 (75.7)	34 (73.9)	0.85
Affected vessels				
1-Vessel affected: no. (%)	41 (17.7)	31 (16.8)	10 (21.7)	0.52
2-Vessels affected: no. (%)	78 (33.8)	67 (36.2)	11 (23.9)	0.12
3-Vessels affected: no. (%)	107 (46.3)	82 (44.3)	25 (54.3)	0.25
Treated vessel/lesion				
Left main: no. (%)	8 (3.5)	5 (2.7)	3 (6.5)	0.20
LAD: no. (%)	87 (37.7)	65 (35.1)	22 (47.8)	0.13
CFx: no. (%)	62 (26.8)	49 (26.5)	13 (28.3)	0.85
RCA: no. (%)	74 (32.0)	62 (33.5)	12 (26.1)	0.38
Coronary artery bypass graft: no. (%)	4 (1.7)	4 (2.2)	0 (0)	0.59
In-stent restenosis: no. (%)	19 (8.2)	14 (7.6)	5 (10.9)	0.55
Aorto-ostial lesion: no. (%)	14 (6.1)	7 (3.8)	7 (15.2)	0.0091
CTO: no. (%)	9 (3.9)	1 (0.5)	8 (17.4)	< 0.001
Further lesion characteristics				
Lesion length: mm	16.0 (10.1, 25.5)	15.0 (10.0, 22.0)	23.7 (12.0, 40.0)	0.0067
True bifurcation lesion: no. (%)	26 (11.4)	14 (7.7)	12 (26.1)	0.0012
Type A lesion: no. (%)	37 (16.0)	35 (18.9)	2 (4.3)	0.013
Type B1 lesion; no. (%)	40 (17.3)	36 (19.5)	4 (8.7)	0.13
Type B2 lesion: no. (%)	41 (17.7)	37 (20.0)	4 (8.7)	0.085
Type C lesion: no. (%)	113 (48.9)	77 (41.6)	36 (78.3)	< 0.001
None/mild lesion calcification: no. (%)	77 (33.3)	62 (33.5)	15 (32.6)	1.00
Moderate lesion calcification: no. (%)	110 (47.6)	95 (51.4)	15 (32.6)	0.031
Severe lesion calcification: no. (%)	44 (19.0)	28 (15.1)	16 (34.8)	0.0054

Categorical variables are presented as counts with corresponding percentages, while continuous variables are summarized using the mean and standard deviation (SD) or the median along with the 25th and 75th percentiles

*CFx* Left circumflex artery; *CTO* Chronic total occlusion; *LAD* Left anterior descending artery; *RCA* Right coronary artery

### Procedural characteristics

Procedural characteristics are displayed in Table 3. Angiographic success was achieved in all treated lesions. Whilst a higher number of drug-eluting stents (DES; 1.0 vs. 2.0; *p*-value < 0.001) was implanted in the manual support population, no differences in the uses of intravascular imaging was noted between the groups. Moreover, a greater proportion of true bifurcation lesions in the manual support group was treated using the provisional technique (8.1% vs. 22.5%; *p*-value = 0.020). Also, longer procedural times (43.0 vs. 67.5 min; *p*-value < 0.001), as well as total contrast fluid volume (130.0 vs. 179.0 mL; *p*-value < 0.001), fluoroscopy times (13.8 vs. 24.4 min; *p*-value < 0.001), and dose-area-products (2218.0 vs. 4071.0 cGycm^2^; *p*-value = 0.002) were documented in procedures requiring manual support. Procedural characteristics stratified according to manual assistance and manual conversion, respectively, are included in Supplementary Table S3.

**Table 3:** Procedural characteristics

	Overall (*N* = 231)	Full robotic (*N* = 185)	Manual support (*N* = 46)	*p*-value
Angiographic success: no. (%)	231 (100)	185 (100)	46 (100)	1.0
Application of blade angioplasty: no. (%)	2 (0.9)	2 (1.1)	0 (0)	1.0
Application of IVL: no. (%)	5 (2.2)	2 (1.1)	3 (6.5)	0.055
Number of DES implanted	1.0 (1.0, 2.0)	1.0 (1.0, 2.0)	2.0 (1.0, 2.8)	< 0.001
Intravascular imaging				
IVUS: no. (%)	44 (19.0)	33 (17.8)	11 (23.9)	0.40
OCT: no. (%)	7 (3.0)	6 (3.2)	1 (2.2)	1.00
Bifurcation technique				
Provisional: no. (%)	23 (10.8)	14 (8.1)	9 (22.5)	0.020
DK-crush: no. (%)	2 (0.9)	0 (0)	2 (5.0)	0.035
Culotte: no. (%)	2 (0.9)	1 (0.6)	1 (2.5)	0.34
Procedural information				
Total procedure time: min	47.0 (33.0, 66.0)	43.0 (29.5, 57.5)	67.5 (51.2, 86.8)	< 0.001
Fluoroscopy time: min	15.2 (10.7, 23.5)	13.8 (9.8, 19.9)	24.4 (18.9, 35.7)	< 0.001
Dose-area-product: cGycm^2^	2326.8 (981.0, 4624.2)	2218.0 (922.8, 4032.2)	4071.0 (1814.0, 6305.1)	0.0019
Total contrast fluid: mL	141.0 (97.0, 190.0)	130.0 (90.0, 180.0)	179.0 (141.0, 215.0)	< 0.001

Categorical variables are presented as counts with corresponding percentages, while continuous variables are summarized using the mean and standard deviation (SD) or the median along with the 25th and 75th percentiles

*DES* drug eluting stents, *IVL* intravascular lithotripsy, *IVUS* intravascular ultrasound, *OCT* optical coherence tomography

### Predictors of manual support, assistance and conversion

**Fig. 1:**
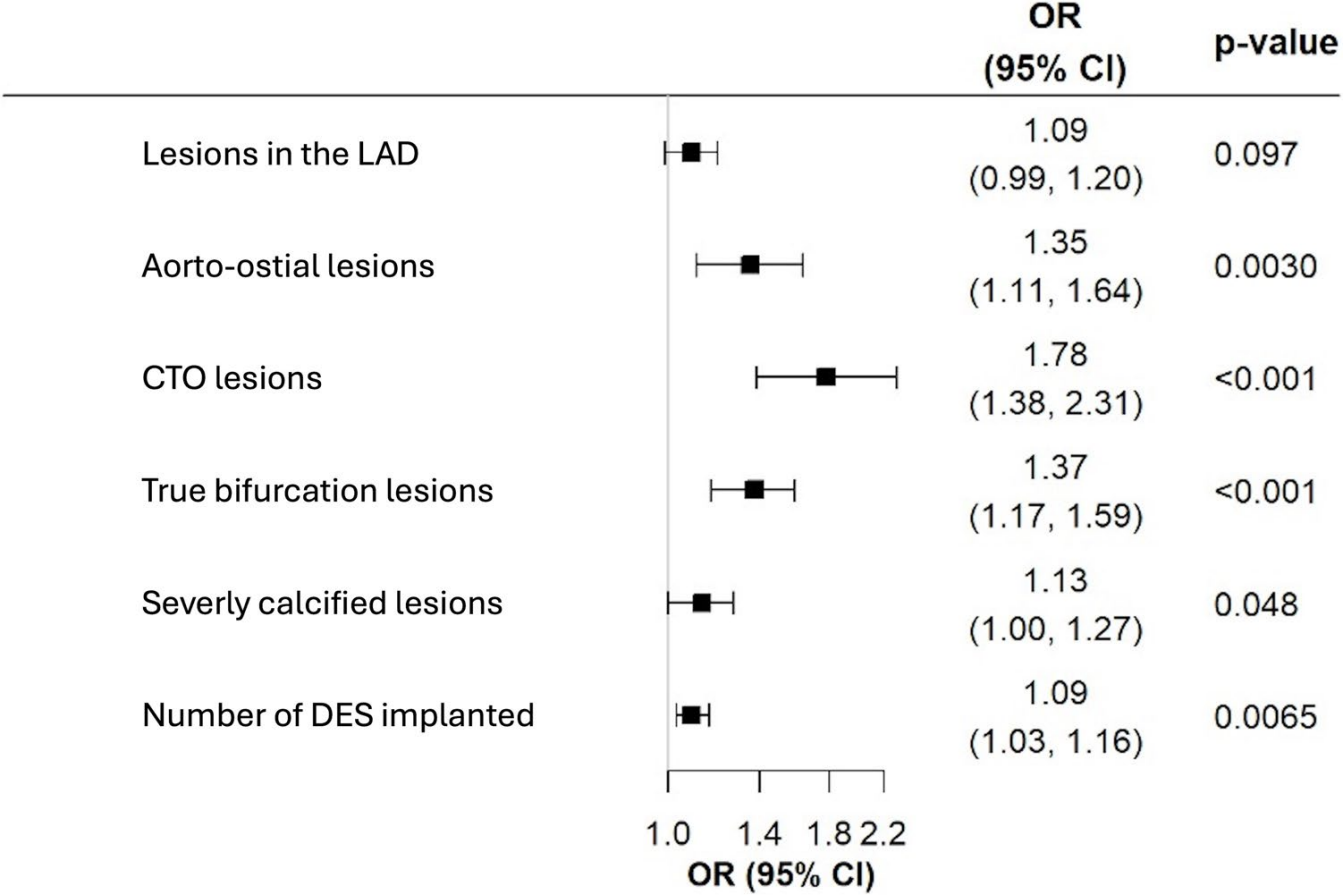
Multivariable predictors of manual support during robotic-assisted percutaneous coronary intervention. Odds ratios (OR) and their 95%-confidence intervals (95%-CI) are provided. *CFx* left circumflex artery, *CTO* chronic total occlusion, *DES* drug eluting stents, *LAD* left anterior descending artery, *RCA* right coronary artery

Univariable predictors of manual support are displayed in Supplementary Table S4. Multivariable stepwise regression analysis identified treated lesions in the LAD [OR: 1.09 (95%-CI: 0.99–1.20)], aorto-ostial lesions [OR: 1.35 (95%-CI: 1.11–1.64)], CTOs [OR: 1.78 (95%-CI: 1.38–2.31)], true bifurcations [OR: 1.37 (95%-CI: 1.17–1.59)], severe calcification [OR: 1.13 (95%-CI: 1.00–1.27)], and the number of implanted DES [OR: 1.09 (95%-CI: 1.03–1.16)] as predictors for manual support (Fig. 1). With regard to the components of manual support, i.e. manual conversion and manual assistance, the following predictors were identified after multivariable regressions: a complete manual conversion was associated with target lesions in the left main trunk [OR: 1.17 (95%-CI: 0.97–1.42)] or LAD [OR: 1.10 (95%-CI: 1.02–1.18)] and severe calcification [OR: 1.11 (95%-CI: 1.01–1.21)], whereas aorto-ostial lesions [OR: 1.32 (95%-CI: 1.14–1.53)], CTO PCIs [OR: 1.75 (95%-CI: 1.43–2.13)], true bifurcation interventions [OR: 1.32 (95%-CI: 1.17–1.48)], and lesion length [per 5 mm increase, OR: 1.02 (95%-CI: 1.01–1.03)] were identified by the model to be predictive for partial manual assistance. All uni- and multivariable predictors of manual assistance and manual conversion, respectively, are displayed in Supplementary Table S5 and S6.

## Discussion

In the current study investigating the need, predictors and implications for manual support during R-PCI in a multi-center cohort, the following main observations were made:

- Nearly one in five patients required some form of manual support during R-PCI.
- Procedures during which manual support was needed were associated with longer procedural times, radiation exposure and contrast fluid volume.
- The identified predictors of manual support mainly represent hallmarks of more complex coronary lesions necessitating the use of advanced interventional techniques.

To the best of our knowledge, the current study represents the first and largest multi-center study investigating manual support during R-PCI. Of note, during the R-PCI procedures included in our study, in a relatively large proportion, i.e. 19.9% of all procedures, some form of manual support was needed. This rate is comparable to the earlier versions of the CorPath system (CorPath 200 System), where in a total of 108 R-PCI procedures 20 (18.5%) involved either planned partial manual assistance (3.7%), unplanned partial manual assistance (7.4%), or a full manual conversion (7.4%) [12]. Of note, for other contemporary robotic PCI platforms, e.g. the R-One robotic system, similar rates of a full manual conversion (3/62 cases, 4.8%) were documented in the multi-center R-EVOLUTION (R-One Efficiency for PCI Evolution With Robotic Assistance) study, whereas the percentage of manual assistance was not reported [13]. Therefore, even with the improved latest generation robotic platforms some form of manual support is needed for a substantial amount of cases. Furthermore, similar to the R-PCI platform employed in our study, the R-One robotic system is also not able to use over-the-wire tools, or is capable to utilize different devices simultaneously, limiting the use of this system in more complex coronary lesions [7].

In the current study, procedural times, contrast volume and radiation exposure were higher in manually supported R-PCI compared with full R-PCI procedures. Probably, this may in particular be explained by the lesion complexity itself leading to more extensive lesion preparation and other interventional challenges, that also require manual support to achieve procedural success. Indeed, the comparison of R-PCI with manual PCI procedures in less complex lesions underline longer procedural times, greater radiation exposure and increased contrast volume use in the R-PCI group [3, 5]. However, in the treatment of more complex lesions by either conventional PCI or R-PCI, differences in dose-area-products and contrast fluid volume were no longer noted in a recent study, thus underscoring that lesion complexity, rather than the use of an R-PCI platform is the most likely driver for differences in procedural characteristics [4].

With regard to the identified predictors of manual support, these variables were largely characteristics of more complex coronary disease, such as true bifurcations, CTOs and calcified lesions. As described above, no currently available R-PCI platform allows simultaneous manipulation of more than one wire/balloon/stent [7, 13]. In addition, the autonomous use of over-the-wire tools, including atherectomy devices and microcatheters, is not possible. Although, the latest CorPath platform used in the current study has improved guide catheter manipulation, also the treatment of aorto-ostial lesions was found to be a predictor for manual support. If the R-PCI technology aims to fulfill its promise of broad utility in both non-complex and complex coronary lesions and utilization for so-called tele-stenting (i.e. PCI performed by a physician with a separate geographic location to the treated patient) further technical advancements of the robotic systems are needed to facilitate robotic, rather than robotic-assisted, PCI in the future. Otherwise, the widespread adoption of R-PCI, comparable to the significant increase in the use of robotics in surgery seems unlikely [14].

## Limitations

The current study builds on a well characterized multicenter cohort of patients undergoing R-PCI, with availability of baseline, lesion and procedural characteristics, enabling insights into the necessity of manual support, assistance and conversion during R-PCI procedures. However, some limitations merit consideration. This study reports from four high-volume PCI centers in Germany, and our findings cannot be readily transferred to other centers. Only a single R-PCI platform was used, and thus no inference of our results to other R-PCI providers/platforms can be made. Lastly, residual confounding with regard to the identified predictors of manual support/assistance/conversion cannot be excluded, although we aimed to limit this by implementing a multivariable stepwise regression analysis.

## Conclusion

In conclusion, in this multi-center analysis, we found manual support was necessary in one out of five patients undergoing R-PCI. In addition, manual support was associated with longer procedural times, radiation exposure and contrast fluid volume. Lastly, strongest predictors for manual support were surrogates for more complex CAD, requiring more advanced PCI techniques. Our results offer further insights into the limitations of current R-PCI platforms and their need for technical refinements in various clinical scenarios.

## Supplementary Information

Below is the link to the electronic supplementary material.

Supplementary file1 (DOCX 34 KB)

## Data Availability

The data underlying this article will be shared on reasonable request to the corresponding author.
