# Supplementary material for: Manual support during robotic-assisted percutaneous coronary intervention
Source: Clin Res Cardiol. 2025 Feb 5;115(1):25–32. doi: 10.1007/s00392-025-02596-6 (PMC12783154; doi:10.1007/s00392-025-02596-6)
Supplement: Supplementary file 1 — Supplementary file1 (DOCX 34 KB) [file 392_2025_2596_MOESM1_ESM.docx]

**Supplementary appendix**

**Supplementary Table S1: Baseline characteristics of patients needing manual assistance or manual conversion during robotic-assisted percutaneous coronary intervention.** Manual assistance was defined as partial manual assistance with the procedure ultimately completed robotically. Manual conversion was defined as bedside manipulation of the guide catheter, guidewire, or delivery system needed to complete the procedure. Categorical variables are presented as counts with corresponding percentages, while continuous variables are summarized using the mean and standard deviation (SD) or the median along with the 25th and 75th percentiles. BMI: Body mass index; eGFR: estimated glomerular filtration rate; LDL-C: Low density lipoprotein cholesterol; LVEF: Left ventricular ejection fraction; PAD: Peripheral arterial disease; PCI: Percutaneous coronary intervention.

|  | **Manual assistance** (N=27) | **Manual conversion** (N=19) | **p-value** |
| --- | --- | --- | --- |
| **Demographics** |  |  |  |
| Age; years | 67.0 (62.0, 78.0) | 70.0 (62.2, 80.8) | 0.54 |
| Male sex; No. (%) | 15 (60.0) | 13 (72.2) | 0.52 |
| BMI; kg/m² | 25.6 (24.3, 28.4) | 25.0 (23.1, 30.8) | 0.78 |
| **Comorbidities and prior medical history** |  |  |  |
| Arterial hypertension; No. (%) | 20 (80.0) | 17 (94.4) | 0.37 |
| Diabetes mellitus; No. (%) | 8 (32.0) | 5 (27.8) | 1.00 |
| Current smoking; No. (%) | 3 (13.0) | 3 (33.3) | 0.31 |
| History of smoking; No. (%) | 15 (60.0) | 11 (61.1) | 1.00 |
| LVEF; % | 50.0 (37.0, 59.0) | 50.0 (40.0, 55.0) | 0.62 |
| History of Cerebrovascular disease; No. (%) | 6 (24.0) | 5 (27.8) | 1.00 |
| History of PAD; No. (%) | 4 (16.0) | 0 (0) | 0.13 |
| History of CABG; No. (%) | 4 (16.0) | 1 (5.6) | 0.38 |
| History of PCI; No. (%) | 18 (72.0) | 8 (44.4) | 0.11 |
| History of myocardial infarction; No. (%) | 9 (36.0) | 7 (38.9) | 1.00 |
| **Laboratory values** |  |  |  |
| LDL-C; mg/dL | 73.5 (59.8, 111.0) | 91.0 (69.0, 113.0) | 0.64 |
| Serum creatinine; mg/dL | 1.0 (0.8, 1.4) | 0.9 (0.8, 1.1) | 0.21 |
| eGFR; ml/min/1.73m^2^ | 71.0 (46.0, 87.9) | 79.6 (67.7, 97.1) | 0.11 |

**Supplementary Table S2: Lesion characteristics of procedures needing manual assistance or manual conversion during robotic-assisted percutaneous coronary intervention.** Manual assistance was defined as partial manual assistance with the procedure ultimately completed robotically. Manual conversion was defined as bedside manipulation of the guide catheter, guidewire, or delivery system needed to complete the procedure. Categorical variables are presented as counts with corresponding percentages, while continuous variables are summarized using the mean and standard deviation (SD) or the median along with the 25th and 75th percentiles. *CFx* Left circumflex artery; *CTO* Chronic total occlusion; *LAD* Left anterior descending artery; *RCA* Right coronary artery.

|  | **Manual assistance** (N=27) | **Manual conversion**  (N=19) | **p-value** |
| --- | --- | --- | --- |
| Radial access; No. (%) | 20 (74.1) | 14 (73.7) | 1.00 |
| **Affected vessels** |  |  |  |
| 1-Vessel affected; No. (%) | 6 (22.2) | 4 (21.1) | 1.00 |
| 2-Vessels affected; No. (%) | 6 (22.2) | 5 (26.3) | 1.00 |
| 3-Vessels affected; No. (%) | 15 (55.6) | 10 (52.6) | 1.00 |
| **Treated vessel/lesion** |  |  |  |
| Left Main; No. (%) | 1 (3.7) | 2 (10.5) | 0.56 |
| LAD; No. (%) | 10 (37.0) | 12 (63.2) | 0.13 |
| CFx; No. (%) | 7 (25.9) | 6 (31.6) | 0.75 |
| RCA; No. (%) | 10 (37.0) | 2 (10.5) | 0.086 |
| Coronary artery bypass graft; No. (%) | - | - | - |
| In-stent restenosis; N No. (%) | 5 (18.5) | 0 (0) | 0.067 |
| Aorto-ostial lesion; N No. (%) | 6 (22.2) | 1 (5.3) | 0.21 |
| CTO; No. (%) | 7 (25.9) | 1 (5.3) | 0.12 |
| **Further lesion characteristics** |  |  |  |
| Lesion length; mm | 29.0 (23.0, 46.9) | 12.0 (8.5, 22.5) | 0.0021 |
| True Bifurcation lesion; No. (%) | 9 (33.3) | 3 (15.8) | 0.31 |
| Type A Lesion; No. (%) | 0 (0) | 2 (10.5) | 0.17 |
| Type B1 Lesion; No. (%) | 2 (7.4) | 2 (10.5) | 1.00 |
| Type B2 Lesion; No. (%) | 3 (11.1) | 1 (5.3) | 0.63 |
| Type C Lesion; No. (%) | 22 (81.5) | 14 (73.7) | 0.72 |
| None/mild lesion calcification; No. (%) | 9 (33.3) | 6 (31.6) | 1.00 |
| Moderate lesion calcification; No. (%) | 10 (37.0) | 5 (26.3) | 0.53 |
| Severe lesion calcification; No. (%) | 8 (29.6) | 8 (42.1) | 0.53 |

**Supplementary Table S3: Procedural characteristics of procedures needing manual assistance or manual conversion during robotic-assisted percutaneous coronary intervention**. Manual assistance was defined as partial manual assistance with the procedure ultimately completed robotically. Manual conversion was defined as bedside manipulation of the guide catheter, guidewire, or delivery system needed to complete the procedure. Categorical variables are presented as counts with corresponding percentages, while continuous variables are summarized using the mean and standard deviation (SD) or the median along with the 25th and 75th percentiles. DES: Drug eluting stents; IVL: Intravascular lithotripsy; IVUS: Intravascular ultrasound; OCT: Optical coherence tomography.

|  | **Manual assistance** (N=27) | **Manual conversion**  (N=19) | **p-value** |
| --- | --- | --- | --- |
| Angiographic success; No. (%) | 27 (100) | 19 (100) | - |
| Application of blade angioplasty; No. (%) | - | - | - |
| Application of IVL; No. (%) | 2 (7.4) | 1 (5.3) | 1.00 |
| Number of DES implanted | 2.0 (1.0, 3.0) | 2.0 (1.0, 2.0) | 0.81 |
| **Intravascular imaging:** |  |  |  |
| IVUS; No. (%) | 8 (29.6) | 3 (15.8) | 0.32 |
| OCT; No. (%) | 0 (0) | 1 (5.3) | 0.070 |
| **Bifurcation technique** |  |  |  |
| Provisional; No. (%) | 7 (33.3) | 2 (10.5) | 0.13 |
| DK-Crush; No. (%) | 1 (4.8) | 1 (5.3) | 1.00 |
| Culotte; No. (%) | 1 (4.8) | 0 (0) | 1.0 |
| **Procedural information** |  |  |  |
| Total procedure time; min | 67.5 (52.2, 86.8) | 70.5 (42.8, 81.5) | 0.53 |
| Fluoroscopy time; min | 23.5 (17.9, 32.4) | 24.4 (20.8, 41.4) | 0.52 |
| Dose-area-product; cGycm^2^ | 4407.0 (1907.0, 6010.0) | 3020.4 (1508.1, 6525.9) | 0.52 |
| Total contrast fluid; mL | 172.0 (136.5, 199.8) | 180.0 (157.0, 250.0) | 0.22 |

**Supplementary Table S4: Univariable predictors of manual support during robotic-assisted percutaneous coronary intervention**. Odds ratios (OR and their 95%-Confidence intervals (95%-CI) are provided. *CFx* Left circumflex artery; *CTO* Chronic total occlusion; DES: Drug eluting stents; *LAD* Left anterior descending artery; *RCA* Right coronary artery.

|  | **OR (95% CI)** | **p-value** |
| --- | --- | --- |
| Treated vessel Left Main | 2.51 (0.50, 10.64) | 0.22 |
| Treated vessel LAD | 1.69 (0.88, 3.26) | 0.11 |
| Treated vessel CFX | 1.09 (0.52, 2.21) | 0.81 |
| Treated vessel RCA | 0.70 (0.33, 1.41) | 0.34 |
| Treated In-stent restenosis | 1.49 (0.46, 4.14) | 0.47 |
| Treated aorto-ostial lesion | 4.56 (1.48, 14.07) | 0.0070 |
| CTO lesion | 38.74 (6.83, 729.39) | <0.001 |
| Treated true bifurcation | 4.26 (1.79, 10.05) | <0.001 |
| Type A Lesion | 1 (reference) |  |
| Type B1 Lesion | 1.94 (0.36, 14.67) | 0.46 |
| Type B2 Lesion | 1.89 (0.35, 14.27) | 0.48 |
| Type C Lesion | 8.18 (2.31, 52.10) | 0.0053 |
| None/mild lesion calcification | 0.96 (0.47, 1.89) | 0.91 |
| Moderate lesion calcification | 0.46 (0.23, 0.89) | 0.025 |
| Severe lesion calcification | 2.99 (1.43, 6.17) | 0.0032 |
| Lesion length, per 5 mm increase | 1.17 (1.07, 1.28) | <0.001 |
| Number of DES implanted | 2.17 (1.49, 3.21) | <0.001 |
| Application of ≥2 coronary wires | 2.55 (1.28, 5.05) | 0.0072 |
| Kissing balloon | 6.19 (2.17, 18.33) | <0.001 |

**Supplementary Table S5: Uni- and multivariable predictors of manual assistance during robotic-assisted percutaneous coronary intervention**. Manual assistance was defined as partial manual assistance with the procedure ultimately completed robotically. Odds ratios (OR and their 95%-Confidence intervals (95%-CI) are provided. *CFx* Left circumflex artery; *CTO* Chronic total occlusion; DES: Drug eluting stents; *LAD* Left anterior descending artery; *RCA* Right coronary artery.

|  | **Univariable Model** | | **Multivariable Model** | |
| --- | --- | --- | --- | --- |
|  | **OR (95% CI)** | **p-value** | **OR (95% CI)** | **p-value** |
| Treated vessel Left Main | 1.08 (0.06, 6.43) | 0.94 |  |  |
| Treated vessel LAD | 0.97 (0.41, 2.19) | 0.94 |  |  |
| Treated vessel CFX | 0.95 (0.36, 2.27) | 0.91 |  |  |
| Treated vessel RCA | 1.29 (0.54, 2.92) | 0.55 |  |  |
| Treated aorto-ostial lesion | 7.00 (2.13, 22.13) | <0.001 | 1.32 (1.14, 1.53) | <0.001 |
| CTO Lesion | 35.35 (7.94, 248.42) | <0.001 | 1.75 (1.43, 2.13) | <0.001 |
| Treated true bifurcation | 5.44 (2.07, 13.85) | <0.001 | 1.32 (1.17, 1.48) | <0.001 |
| None/mild lesion calcification | 1.00 (0.41, 2.29) | 1.00 |  |  |
| Moderate lesion calcification | 0.61 (0.26, 1.38) | 0.24 |  |  |
| Severe lesion calcification | 1.96 (0.76, 4.71) | 0.14 |  |  |
| Lesion length, per 5 mm increase | 1.27 (1.15, 1.41) | <0.001 | 1.02 (1.01, 1.03) | <0.001 |
| Number of DES implanted | 2.15 (1.39, 3.32) | <0.001 |  |  |
| Application of ≥2 coronary wires | 3.81 (1.66, 8.78) | 0.0015 |  |  |
| Kissing balloon | 7.58 (2.48, 22.63) | <0.001 |  |  |

**Supplementary Table S6: Uni- and multivariable predictors of manual conversion during robotic-assisted percutaneous coronary intervention**. Manual conversion was defined as bedside manipulation of the guide catheter, guidewire, or delivery system needed to complete the procedure. Odds ratios (OR and their 95%-Confidence intervals (95%-CI) are provided. *CFx* Left circumflex artery; *CTO* Chronic total occlusion; DES: Drug eluting stents; *LAD* Left anterior descending artery; *RCA* Right coronary artery.

|  | **Univariable Model** | | **Multivariable Model** | |
| --- | --- | --- | --- | --- |
|  | **OR (95% CI)** | **p-value** | **OR (95% CI)** | **p-value** |
| Treated vessel Left Main | 4.04 (0.56, 19.15) | 0.10 | 1.17 (0.97, 1.42) | 0.097 |
| Treated vessel LAD | 3.13 (1.21, 8.73) | 0.022 | 1.10 (1.02, 1.18) | 0.010 |
| Treated vessel CFX | 1.29 (0.43, 3.42) | 0.63 |  |  |
| Treated vessel RCA | 0.23 (0.04, 0.83) | 0.053 |  |  |
| Treated aorto-ostial lesion | 0.85 (0.05, 4.66) | 0.88 |  |  |
| CTO Lesion | 1.42 (0.07, 8.37) | 0.75 |  |  |
| Treated true bifurcation | 1.52 (0.34, 5.02) | 0.53 |  |  |
| None/mild lesion calcification | 0.92 (0.31, 2.42) | 0.87 |  |  |
| Moderate lesion calcification | 0.36 (0.11, 0.99) | 0.061 |  |  |
| Severe lesion calcification | 3.56 (1.30, 9.42) | 0.011 | 1.11 (1.01, 1.21) | 0.028 |
| Lesion length, per 5 mm increase | 0.92 (0.74, 1.07) | 0.35 |  |  |
| Number of DES implanted | 1.59 (0.94, 2.59) | 0.070 | 1.03 (0.99, 1.08) | 0.14 |
| Application of ≥2 coronary wires | 1.04 (0.33, 2.87) | 0.94 |  |  |
| Kissing balloon | 1.66 (0.25, 6.64) | 0.52 |  |  |
